# The effect of moderate- and high-intensity interval training on substrate oxidation and nutrient preferences in obese men

**DOI:** 10.1186/1550-2783-11-S1-P7

**Published:** 2014-12-01

**Authors:** Shaea A Alkahtani

**Affiliations:** 1 University of Dammam, Dammam, Saudi Arabia

## Background

This study compared the influence of 12 sessions of moderate- and high-intensity interval training on substrate oxidation and nutrient preferences.

## Methods

Ten obese men participated in cross-over 4-week moderate- (MIIT) and high- (HIIT) intensity interval cycling. MIIT consisted of 5-min stages at ±20% of mechanical work at 45 %VO_2peak_, and HIIT consisted of 30-sec work at 90 %VO_2peak_ and 30-sec rests for 30-45 min. The assessments included a constant-load test consisted of 5-min rest, 45-min cycling at 45 %VO_2peak_ followed by 60-min recovery. Intermittent measures of gas exchange using indirect calorimetry were undertaken, and *ad libitum* meal was provided after the test. Consent to publish the results was obtained from all participants.

## Results

Changes in fat oxidation were +19% at rest, +96% during exercise and +59% during recovery in MIIT, and were -7% at rest, +43% during exercise and -13% during recovery in HIIT. Changes in CHO oxidation were +6% at rest, -13% during exercise and -2% during recovery in MIIT, and were +46% at rest, -7% during exercise and +32% during recovery in HIIT. The amount of meal, fat and CHO eaten decreased by 24, 16 and 13% respectively after HIIT, and increased by 3, 38 and 14% respectively after MIIT. The intervention explained 12.4% of the changes in fat intake (p = 0.07). The interaction of fat oxidation*intervention did not significantly explain the change in fat eaten (p>0.05).

## Conclusion

The change in fat oxidation did not explain the change in fat eaten after interval training.

